# MAO-B elevation decreases parkin's ability to efficiently clear damaged mitochondria: protective effects of rapamycin

**DOI:** 10.3109/10715762.2012.662277

**Published:** 2012-02-14

**Authors:** Almas Siddiqui, Ingrid Hanson, Julie K. Andersen

**Affiliations:** Buck Institute for Research in Aging, Novato, CA, USA

**Keywords:** parkin, mTOR, rapamycin, oxidative stress, monoamine oxidase-B, mitophagy

## Abstract

Increased oxidative stress in the Parkinsonian substantia nigra is believed to contribute to neurodegeneration, in part due to regionally elevated levels of the enzyme monoamine oxidase B (MAO-B). Increased oxidative stress has also been reported to be associated with the inhibition of E3 ligase activity of the Parkinson's disease-related protein parkin. In an inducible MAO-B cell model, losses in parkin E3 ligase activity were found to occur in conjunction with reduced mitochondrial turnover and decreased mitochondrial function, although this did not inhibit parkin's ability to translocation to damaged mitochondria. The mTOR inhibitor rapamycin was found to restore both mitophagy and mitochondrial function in these cells. These data suggest that MAO-B induction can interfere with mitochondrial quality control via losses in parkin activity that in turn impact on mitochondrial turnover. Rapamycin may be an effective means of counteracting the effects of lost parkin function by independently enhancing autophagic removal of damaged mitochondria.

## Introduction

Studies in both post-mortem patient tissues and Parkinsonian animal models have provided strong evidence supporting the involvement of oxidative stress in the progression of Parkinson's disease (PD). One possible source of increased oxidative stress are elevations in brain monoamine oxidase B (MAO-B) levels, which have been demonstrated to increase with age and in association with neurodegenerative disease both in humans and in mice [12,13]. Substrate oixdation by MAO-B is accompanied by the stoichiometric reduction of oxygen to hydrogen peroxide (H_2_O_2_) [5,17]. It has been postulated that age-related increases in MAO-B activity may contribute to cellular neurodegeneration in the brain due to corresponding increases in H_2_O_2_ production [10]. MAO-B catalysed ROS production has also been suggested to contribute to age-related increases in mitochondrial damage, particularly in the substantia nigra (SN), a brain region, which displays preferential neurodegeneration in PD [19]. Mitochondrial dysfunction has long been implicated in the pathogenesis of idiopathic PD [8].

In order to assess the potential role of MAO-B- induced ROS in associated mitochondrial dysfunction, we created both PC12 cell lines [23] and transgenic mice in which MAO-B levels could be specifically induced [20]. We discovered that elevations in MAO-B activity in these models at concentrations found to be associated with aging and disease resulted in significantly increases in levels of mitochondrial dysfunction as a consequence of increased H_2_O_2_ generation [8,18]. However, the possible mechanisms involved were not fully elucidated.

Parkin is ubiquitin E3 ligase, which has recently been reported to be recruited to impaired mitochondria and to promote their autophagic degradation via the lysosome [3]. When parkin becomes dysfunctional, the clearance of damaged mitochondria is prevented resulting in overall cellular mitochondrial dysfunction [4]. Mutations in the parkin (PARK2) gene in certain forms of familial PD have been associated with functional loss of the protein's E3 ligase activity [6]. Many of these mutations appear to alter the solubility of parkin, resulting in the formation of cytoplasmic parkin aggregates that may account for its enzymatic inhibition. In addi-tion to genetic mutations, exposure to oxidative stress has also been reported to result in the forma-tion of insoluble parkin aggregates and decreased E3 ligase activity [11].

Here, we report that induction in human MAO-B levels *in vitro* to levels associated with sporadic forms of PD results in reductions in parkin's E3 ligase activity. These reductions did not prevent the protein from being recruited to damaged mitochondria, but did result in an accumulation of damaged mitochon-dria and an overall cellular reduction in mitochondrial function. Co-treatment of cells with the mTOR inhibitor rapamycin was found to promote clearance of damaged mitochondria and to rescue losses in mitochondrial deficits elicited by MAO-B increase.

## Methods

All chemicals were from Sigma unless otherwise noted.

### Generation of inducible MAO-B cell lines constitutively-expressing human parkin

A stable doxycycline (dox)-inducible human MAO-B PC12 cell line previously generated and characterized by the Andersen laboratory [9] was used for the current studies. MAO-B cells were transfected with 3X-FLAG vector containing an insertion of human parkin cDNA (gift of Keio University School of Medicine Tokyo, Japan) and a neomycin selection marker (Clontech) using Lipofectamine 2000 reagent (Invitrogen). Parkin-containing clones were selected via growth at 37°C in Dulbecco's modified Eagle's medium (DMEM) containing 10% Tet-FBS (Clontech), 5% horse serum (GIBCO), 1% streptomycinpenicillin (GIBCO) and 200 μg/ml of G418 (Cellgro). Transfection efficiency was determined via Western blot analysis using an anti-mouse 3X-FLAG antibody (Sigma–Aldrich). Cells were differentiated into neural cells using 50 ng/ml of nerve growth factor (NGF) (Sigma–Aldrich) administered one day prior to dox treatment.

### Doxycycline, FCCP and rapamycin treatment

Oxidative stress conditions were induced via treatment with dox (40 μg/ml, Sigma–Aldrich) for 16 hours to stimulate MAO-B expression [9]. Non-toxic rapamycin concentrations used for described studies were initially determined via cell viability analyses using the 3-(4,5-dimethylthiazol-2-yl)-2,5-diphenyltetrazolium bromide (MTT) assay. Cells were pre-treated with rapamycin (Sigma–Aldrich) for 1 hour prior to dox treatment. For experiments involving FCCP, cells were treated with 4 μM of the agent for 1 hour following dox treatment in the absence or presence of rapamycin prior to cell collection.

### E3 ligase activity assay via assessment of parkin auto-ubiquitination

Immunoprecipitation of transgenic human parkin protein was carried out using Dynabeads® anti-Mouse IgG magnetic beads (Invitrogen). Cellular protein fractions were pre-cleared of non-specific IgG antibody using non-antibody bound beads for 1 hour at 4°C. While samples were being pre-cleared, 3X-FLAG antibody was bound to magnetic beads via incubation at 4°C for 2 hours. Pre-cleared samples were then immunoprecipitated using 3X-FLAG antibody for 2 hours. Samples were incubated in E3 ligase cocktail (2 mM ATP, 50 mM Tris-Cl, pH 7.4, 2.5 mM MgCl_2_, 100 ng E1 ligase and 250 ng E2 ligase) either with or without 10 μg ubiquitinCH5 (UbCH5) for 2 hours. Parkin auto-ubiquitination was analysed by subsequent immunoblot analysis (see below) using parkin or ubiquitin antibodies; actin was used as a loading control.

### Mitochondrial fractionation

Mitochondrial fractionation was carried using the QIAGEN Qproteome™ mitochondria isolation kit. All buffers and solutions used in this protocol were supplemented with protease inhibitor. After collection in PBS, cell suspensions were centrifuged at 4°C at 300 × g for 3 minutes, followed by the removal of supernatant. The cell pellet was then re-suspended in ice-cold lysis buffer for 10 minutes at 4°C followed by centrifugation at 1000 × g for 10 minutes at 4°C. The resulting supernatant was removed and retained as the cytoplasmic fraction. The cell pellet was resuspended in ice-cold disruption buffer and then centrifuged at 1000 × g for 10 minutes at 4°C. The resulting supernatant was centrifuged at 6000 × g for 10 minutes at 4°C.The resulting whole mitochondrial pellet was washed in mitochondrial storage buffer and centrifuged at 6000 × g for 20 × minutes at 4°C. The resulting supernatant was discarded and the mitochondrial pellet re-suspended in mitochondrial storage buffer for use in subsequent assays.

### Immunoblot analyses

For whole cell westerns, cells were lysed in RIPA buffer [50 mM Tris, pH 7.4, 1% Igepal, 0.25% sodium deoxycholate, 150 mM NaCl, 1 mM EDTA, and a proteinase inhibitor cocktail (Roche Molecular Biochemicals)]. After centrifugation at 15,000 × g for 15 minutes at 4°C, whole cell lysate was collected from the resulting supernatant and protein content determined using the Bradford method [2]. Mitochondrial fractions or whole cell protein samples were run on 4–12% or 10% NuPAGE® Bis-Tris Gels (Invitrogen) followed by the transfer of protein to polyvinyl difluoride membranes (Immobilon). Membranes were blocked with a 5% blocking solution (5% powdered milk dissolved in 0.1% Tween/phosphate buffered saline) prior to incubation with 3X-FLAG (1:1000 dilution; Sigma Aldrich), parkin (1:1000 dilution; Millipore), or ubiquitin (1:1000 dilution; Dako Cytomation) antibodies; Negative reaction controls included incubation of tissues in the absence of primary antibody. β-Actin (1:5000 dilution; Millipore) or VDAC1 (1:1000 dilution;Abcam) antibodies were used as whole cell or mitochondrial protein loading controls, respectively. Membranes were incubated in corresponding HRP-conjugated secondary antibodies (Santa Cruz) for 1 hour following incubation with primary antibody. Protein bands were detected using chemiluminescence substrate for horse-radish peroxidase (HRP) (Amersham Biosciences, Millipore). Resulting bands were captured on Kodak BioMax Light film, developed, scanned and densitometry measured and quantified using the NIH Image J application.

### Immunocytochemistry (ICC)

To assess mitochondrial parkin and lysosomal LC3 expression, cells were seeded in 8-chamber slides (Thermo Scientific/Nunc), treated, incubated with 10 μM Mitotracker Red® (Invitrogen) for 20 minutes, and then fixed with 4% paraformaldehyde solution (PFA) for 20 minutes. Once fixed, cells were washed twice with PBS. Fixed cells were then blocked with 5% normal donkey serum (NDS) for 1 hour at room temperature followed by an overnight incubation with primary antibodies (parkin or LC3) at 4°C. Cells were then incubated with Alexa-conjugated fluorescent secondary antibodies (Invitrogen) for 1 hour in the absence of light at room temperature to allow the fluorescent detection of desired proteins. Images were captured using a Zeiss LSM 510 confocal microscope.

### Mitochondrial respiration assays via the XF24 Seahorse microrespirometry

For these experiments, cells were seeded onto 24-well Seahorse microplates (40,000 cells per well) prior to analysis in the Seahorse XF24. All experiments were conducted at 37°C. Wells containing an estimated volume of 500 μl of Seahorse media (3.5 mM KCl, 120 mM NaCl, 1.3 mM CaCl_2_-2H_2_O, 0.4 mM KH_2_PO_4_, 1.2 mM Na_2_SO_4_, 15 mM D-Glucose, 2 mM MgSO_4_ and 10 mM TES) were analysed for mean oxygen consumption rates (basal respiration, following addition of 4 μg/ml oligomycin to assess proton leak, and 6 μm FCCP for maximal respiration). The GraphPad Prism program was used for statistical analysis of respiration measurements using the Tukey–Kramer method.

### Mitochondrial complex I activity

Complex I activity was assayed in isolated mitochondrial fractions as rotenone-sensitive NADH dehydrogenase activity as previously described [24]. Values were normalized per protein using Bradford Assay.

### Statistics

One-way ANOVA followed by Newman–Keuls Multiple comparison was used to compare differences between groups unless otherwise noted.

## Results

### MAO-B induction results in decreases in parkin's ubiquitin E3 ligase activity

**Figure 1:**
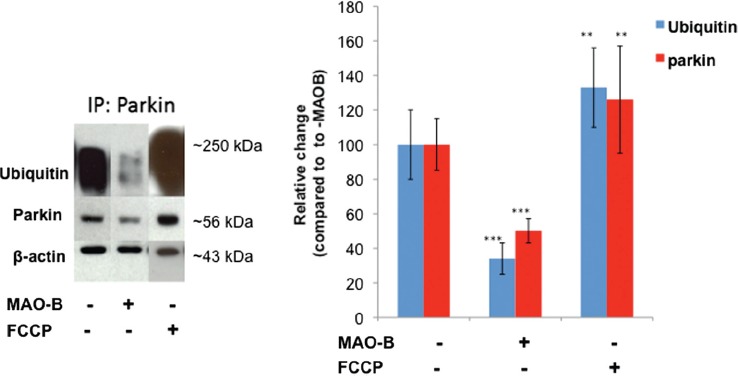
Decreased parkin ubiquitin E3 ligase activity following MAO-B induction. Inducible MAO-B PC12 cells were stably transfected with human parkin and assessed under both control and dox-induced conditions versus following FCCP treatment. Cell lysates were prepared from cells and immunoprecipitated (IP) using Flag-tagged or parkin antibody. Samples were run in an E3 ligase activity assay followed by analysis via Western blot using parkin and ubiquitin antibodies; β-actin was used as loading control. Corresponding densitometric analyses are shown (n = 3). Values are expressed as mean ± SD; ^***^ *p* < 0.001 versus parkin–MAO-B cells, ^**^ *p* < 0.01 versus × MAO-B cells.

We first assessed whether MAO-B elevation had effects on parkin E3 ligase function that could prevent the enzyme from ubiquitin-tagging dysfunctional mitochondria for lysosomal degradation. To monitor E3 ligase activity, we assessed parkin autoubiquitination in dox-inducible MAO-B cells stably transfected with human parkin in the absence and presence of dox or FCCP-induced mitochondrial depolarization (Figure 1). MAO-B-induced oxidative stress was found to negatively impact on parkin's ability to ubiquitinate itself. Mitochondrial depolarization via FCCP in contrast was not found to inhibit E3 ligase activity.

### Parkin's translocation to mitochondria increases in response to MAO-B induction and is exacerbated in cells treated with FCCP and/or rapamycin

**Figure 2:**
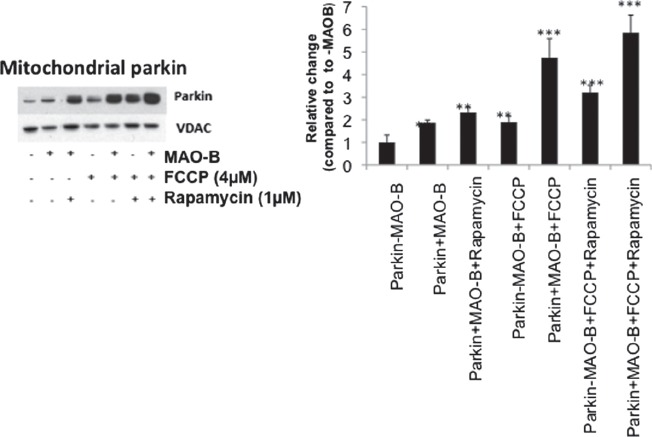
Mitochondrial parkin levels in various conditions. Western blot analyses were performed on mitochondrial sub-fractions from cells in various treatment conditions using Flag or parkin antibodies (Sigma). Immunoblots were normalized for VDAC (Chemicon) for corresponding densitometric analyses (n = 3). Values are expressed as mean ± SD, ^**^ *p* < 0.01 ^***^ *p* < 0.001 versus –MAO-B cells.

Parkin recruitment to damaged mitochondria has been reported to increase in response to depolarization by FCCP (31). Direct oxidative damage of mitochondria (such as that induced by MAO-B increase) may also result in their depolarization and subsequent parkin recruitment. In the present study, immunoblot analysis of whole mitochondrial fractions isolated from MAO-B-parkin cells revealed increased mitochondrial parkin levels in response to both MAO-B-induced oxidative stress and depolarization via FCCP and in combination were found to be additive (Figure 2). Rapamycin treatment in the context of either MAO-B induction or depolarization also appeared to result in increased mitochondrial parkin levels, which were also additive in combination.

**Figure 3:**
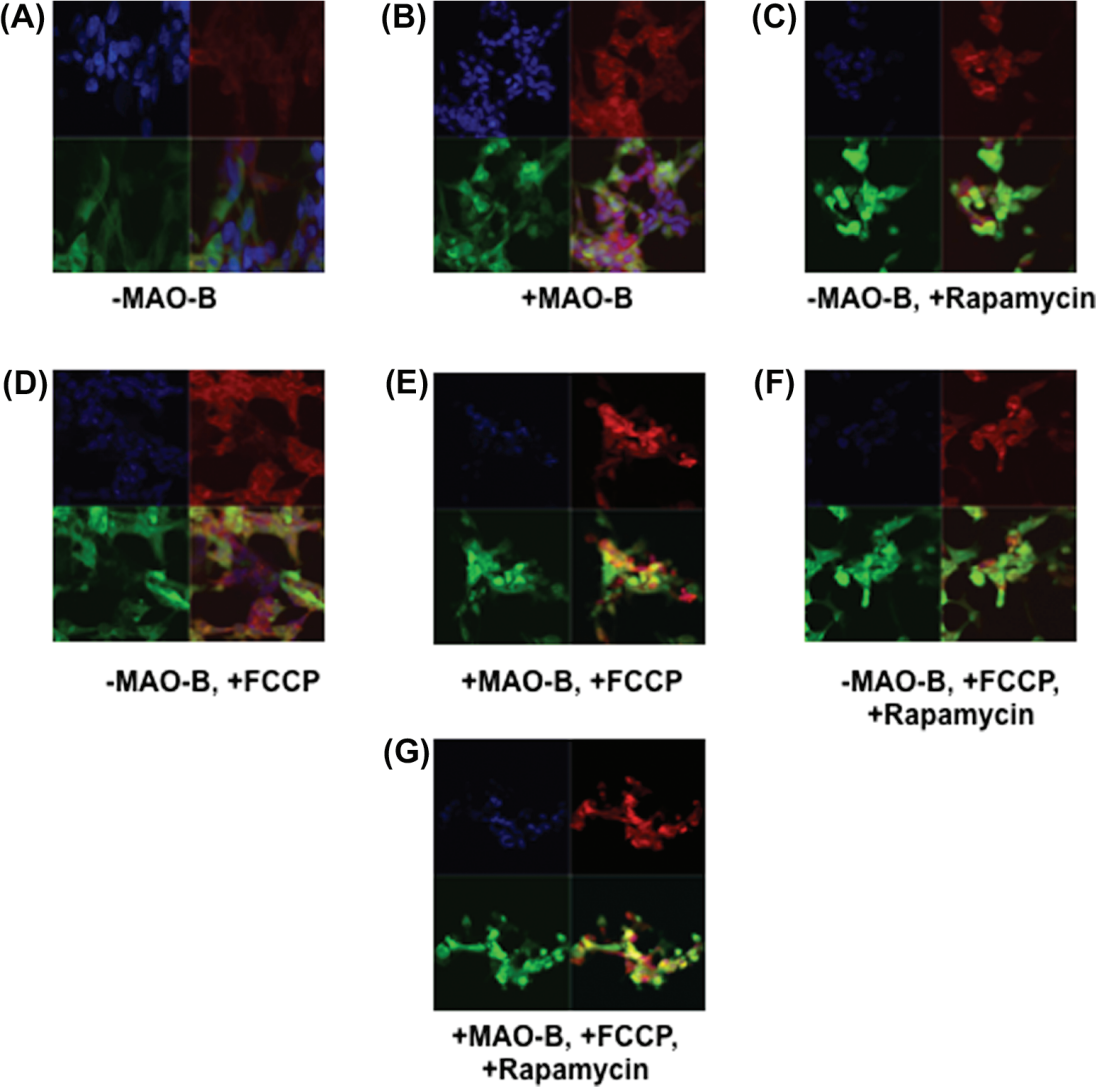
Immunocytochemistry (ICC) analysis of mitochondrial localization of parkin in various conditions. Z-stack images of parkin (green), mitotracker red (red), DAPI (blue), and merged (yellow) were captured using LSM510 confocal microscopy, white arrows indicating formation of yellow punctae. (A)–MAO-B, (B) ⊞ MAO-B, (C) –MAO-B, 1 μM rapamycin pre-treatment, (D) – MAO-B, 4 μM FCCP treatment, (E) ⊞ MAO-B, 4 μM FCCP treatment, (F) −MAO-B, ⊞ FCCP, ⊞ rapamycin, (G) ⊞ MAO-B, ⊞ FCCP, ⊞ rapamycin.

**Figure 4:**
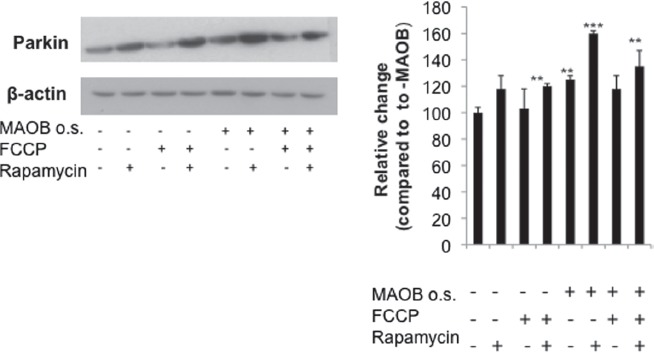
Cellular parkin levels in various conditions.Whole cell lysates fractions were analysed via Western blot analysis using flag antibody (Sigma); β-actin was used as loading control (Chemicon). Corresponding densitometric analyses are shown (n = 3). Values are expressed as mean ± SD; ^***^ *p* < 0.001 ^**^ *p* < 0.01 versus –MAO-B cells.

To validate whether increases in mitochondrial parkin levels were due to increased localization of the protein to this organelle, parkin-MAO-B cells were examined using fixed cell imaging. Higher expression of parkin (green) within mitochondria (labelled with mitotracker red), resulting in increases in merged yellow staining was used as a marker of mitochondrial parkin localization. Mitochondrial parkin localization was found to increase in response to MAO-B induction and to be further exacerbated in the presence of FCCP, correlating with the immunoblot data (Figure 3). Rapamycin also appeared to result in increased mitochondrial parkin protein levels; elevations in overall cellular parkin also appear to occur in response to either MAO-B induction or rapamycin treatment (Figure 4).

### Mitophagy is inhibited in response to MAO-B induction but restored in the presence of rapamycin treatment

**Figure 5:**
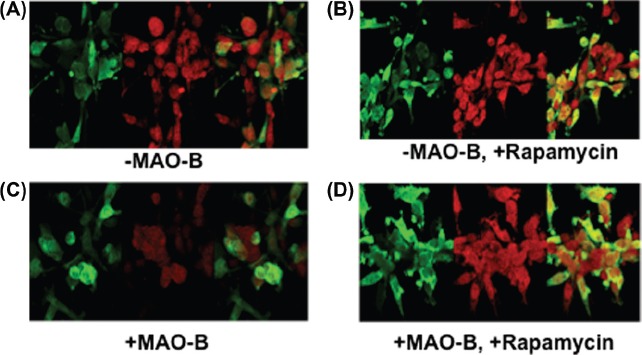
Z-stack images of parkin (green), LC3 (red), and merged (yellow) were taken using LSM 510 confocal microscopy. (A) –MAO-B; (B) ⊞ MAO-B; (C) –MAO-B, 1 μM rapamycin; (D) ⊞ MAO-B, 1 μM rapamycin. Images were taken at the following parameters: (x) 126.8 μm x (y) 140.6 μm x (z) 7.0 μm.

Although increased MAO-B induction appears to result in increased translocation of parkin to the mitochondria, the protein may not be functionally capable of tagging damaged mitochondria for lysosomal removal due to corresponding losses in parkin ubiquitin E3 ligase activity. We examined the effects of MAO-B induction on mitophagy and whether this process was impacted by pre-treatment with rapamycin. To perform these studies, parkin-MAO-B cells were fixed and analysed for alterations in the autophagic marker LC3 and parkin expression in the various cellular conditions. Our experiments demonstrated a decrease in LC3 following induction of human MAO-B expression, suggesting decreased efficiency of autophagic degradation (Figure 5). This data was further verified by Western blot analysis of LC3 levels (data not shown). In agreement with data from other laboratories (1, 14, 21), pre-treatment with rapamycin was found to increase LC3 expression, suggesting restoration of mitophagy reduced as a consequence of MAO-B induction.

### MAO-B induction results in a loss in spare capacity and mitochondrial complex I activity which are restored by rapamycin treatment

**Figure 6:**
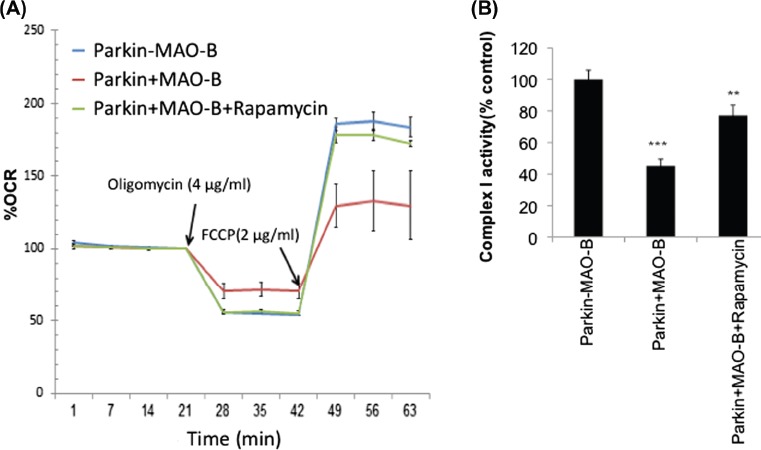
(A) Mitochondrial respiration measurements reported as percent oxygen consumption rate (OCR) versus –MAO-B controls in the absence or presence of oligomycin and FCCP application (at time of arrow) ⊞ /–rapamycin pre-treatment. (B) Mitochondrial complex I activity reported as percent –MAO-B controls. Values are expressed as mean ± SD; ^***^ *p* < 0.01 ^**^ *p* < 0.001 compared to –MAO-B cells.

Reductions in mitophagy could result in subsequent reductions in overall cellular metabolic function. Levels of overall mitochondrial respiration in response to MAO-B in cells expressing human parkin were therefore analysed using the Seahorse XF Extracellular Analyzer. Basal respiration rates were measured followed by measurement of respiration in the presence of oligomycin and FCCP to assess ATP synthesis efficiency and maximal respiratory capacity, respectively. Results from these studies (Figure 6) demonstrate that MAO-B induction results in effects on both oligomycin-sensitive respiration and maximal respiratory rates that were reversed in the presence of rapamycin treatment. Human parkin expression alone appeared to have no significant effects on either (data not shown). Mitochondrial complex I activity, another measure of mitochondrial function, was also found to be inhibited in the presence of MAO-B induction and restored by rapamycin.

## Discussion and conclusions

Major findings from this study suggest that, under con-ditions of MAO-B induction related to aging and PD, parkin's E3 ligase activity is inhibited. This is in agreement with previous studies demonstrating that application of exogenous oxidants such as H_2_O_2_ result in reductions in parkin solubility, formation of insoluble aggregates and loss of E3 ligase activity [11,19]. Parkin is still, however, able to translocate to the mitochondria; indeed this ability appears to be heightened particularly under conditions of mitochondrial damage and depolarization (MAO-B, FCCP), suggesting that mitochondrial recruitment of the protein is separate from its enzymatic function. However, losses in parkin's E3 ligase activity appears to have marked effects on its ability to participate in mitochondrial turnover. The homeostatic turnover of damaged mitochondria is essential to cellular maintenance, as these organelles are recognized as master regulators of cellular function via their involvement in both energy production and the promotion of cell death via apoptosis [7,22]. Accumulation of damaged mitochondria within affected cells can lead to cellular dysfunction; we observed in our studies that reductions in lysosomally-mediated mitochondrial clearance as a consequence of elevations in MAO-B correlated with decreased efficiency of ATP synthesis, spare respiratory capacity, and mitochondrial complex I activity. The mTOR inhibitor rapamycin was able to counteract the effects of loss of parkin function via increased mitophagy, resulting in improved cellular metabolic function.

An interesting finding in this study is the noted increase in parkin protein levels in response to MAO-B induction. We speculate that this may a compensatory response to the presence of associated elevations in mitochondrial damage, but likely abortive in the context of chronic on-going oxidative stress and continued inhibition of parkin E3 ligase activity. We also noted an increase in parkin protein levels in the context of rapamycin treatment, particularly in the presence of MAO-B or FCCP–mediated mitochondrial stress. Its up-regulation in this context could be a consequence of increased levels of the downstream mTOR target 4EBP1 [25]. 4EBP1 levels are increased following mTOR inhibition and have been demonstrated to sub-sequently increase the translation of several mitochondrial proteins [25]. Whether parkin is a target of this action of 4EBP1 is currently under investigation in our laboratory. We hypothesize, however, that rapamycin's ability to increase parkin levels in stressed cells is also likely to be an abortive compensatory event and that its neuroprotective affects are more likely due to its ability to prevent mTOR-mediated repression of lysosomal autophagy independent of parkin.

In the case of PD, mitochondrial dysfunction is believed to occur in response to accelerated rates of oxidative stress although the mechanisms involved have yet to be completely elucidated [7]. Our data suggests that losses in cellular mitochondrial function as a consequence of MAO-B induction may be at least in part due to effects on oxidative effects on parkin activity that impact on its ability to remove damaged mitochondria and preserve overall cellular metabolic function. Many recent therapeutic studies have indeed focused on drugs that can restore the proper turnover of damaged mitochondria including rapamycin. Rapamycin appears to promote the degradation of both aggregated proteins and damaged mitochondria [1] and has been shown to exhibit neuroprotective properties in several disease models including rotenone treatment [1,14–16]. Rapamycin treatment has recently been shown to prevent mitochondrial defects and neuronal death in cell lines derived from parkin-deficient PD patients [21]. Our studies suggest that rapamycin may be an effective means of counteracting the effects of loss in parkin function by allowing cells to regain the ability to facilitate the removal of damaged mitochondria.

In conclusion, clarifying the mechanistic pathways through which oxidative stress including that induced by MAO-B induction alters parkin function and the process of parkin-mediated mitophagy will aid in the understanding hallmarks associated with autosomal recessive forms of PD. Elucidation of parkin dysfunction induced by oxidative stress may in addition one day facilitate a better understanding of the more common sporadic form of PD and provide mechanistic insights that will aid in the development of new therapeutic agents for the treatment of PD.
